# A rare variation of hydranencephaly: case report

**DOI:** 10.12688/f1000research.1-22.v3

**Published:** 2014-01-30

**Authors:** Buddhika TB Wijerathne, Geetha K Rathnayake, Sisira K Ranaraja

**Affiliations:** 1 Obstetrics and Gynaecology Unit, Teaching Hospital Peradeniya, Peradeniya, Sri Lanka; 2 Current address: Department of Forensic Medicine, Faculty of Medicine and Allied, Rajarata University of Sri Lanka, Saliyapura, Sri Lanka; 3 Obstetrics and Gynaecology Unit, Castle Street Hospital for Women, Colombo, Sri Lanka; 4 Current address: Teaching Hospital Anuradhapura, Anuradhapura,, Sri Lanka

## Abstract

Hydranencephaly is a rare congenital abnormality characterized by the absence and replacement of the cerebral hemispheres with cerebrospinal fluid. Here, we present an ultrasonographic diagnosis of a case of a rare variant of fetal hydranencephaly at 38 weeks of gestation. Obstetric sonography revealed the absence of the cerebral cortex, thalami and basal ganglia with a disrupted falx and preserved posterior fossa structures. This is the first reported case of hydranencephaly with the absence of the thalami and basal ganglia, which was diagnosed prenatally. The diagnosis was confirmed with postnatal computed tomography. The early prenatal diagnosis allowed for prompt obstetric attention at a tertiary care hospital which had specialized pediatric facilities including prenatal counseling and support.

## Introduction

Hydranencephaly is a rare congenital abnormality characterized by the absence and replacement of the cerebral hemispheres with cerebrospinal fluid. It was first described by Cruveilher (1892) as “Anencephalie hydrocephalique” or “Hydroanencephalie” ^1^. Crome and Sylvester then reviewed the disease and defined it as a congenital condition ^2^. It is an extremely rare and unique abnormality occurring in less than 1 per 10,000 births worldwide ^3^. It is also said to be present in 0.2% of infant autopsies, and approximately 1% of babies diagnosed clinically as hydrocephalus ^4^. It is critical to differentiate between hydranencephaly and extreme hydrocephalus as the latter carries a potentially better prognosis ^5^. Here, we report a rare case of hydranencephaly, which was diagnosed in the late third trimester, followed by successful obstetric care and management.

## Case presentation

A 28-year-old women (Gravida 2 and para 1) at 38 weeks of gestation was presented to our obstetrics and gynecology unit with lower abdominal pain. She and her husband were Sinhalese and unrelated, with no family history of genetic or congenital anomalies. The patient didn’t smoke, and there was no history that suggested congenital infections or exposure to toxins. In her first pregnancy she delivered a normal healthy male child by an uncomplicated vaginal delivery. In her most recent pregnancy, she didn’t attend the local antenatal clinic regularly, and thus was recognized as a noncompliant patient. She had not had a routine dating scan, anomaly scan or growth scan of the fetus during her pregnancy. The clinical obstetric examination at presentation was unremarkable, and the cardiotocography of the fetus was normal.

**Figure 1.**
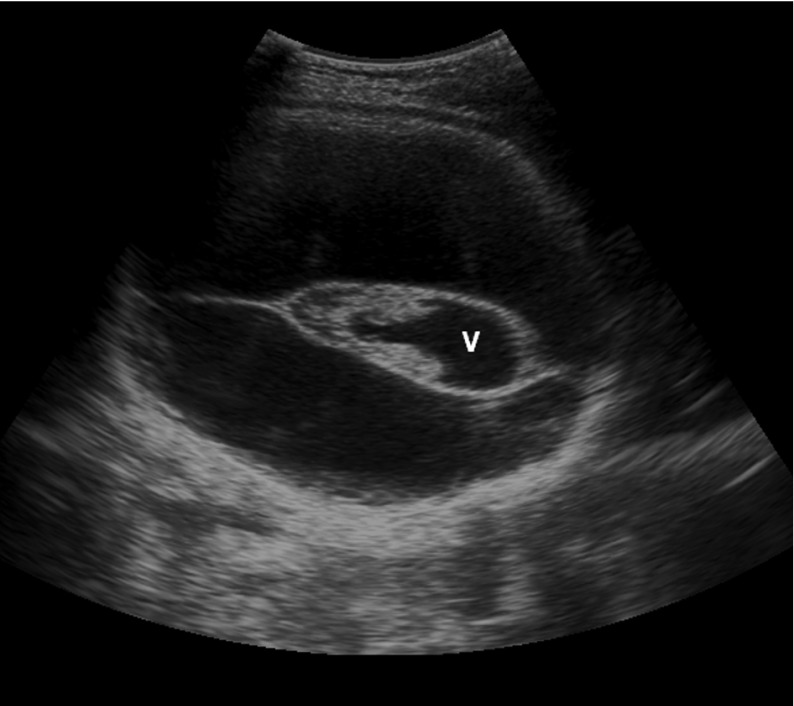
Prenatal ultrasound scans image of the fetus. Transabdominal transverse section of the fetal head at 38 weeks of gestation shows dilated third ventricle (V) with absent thalami and basal ganglia and cerebral cortex.

**Figure 2.**
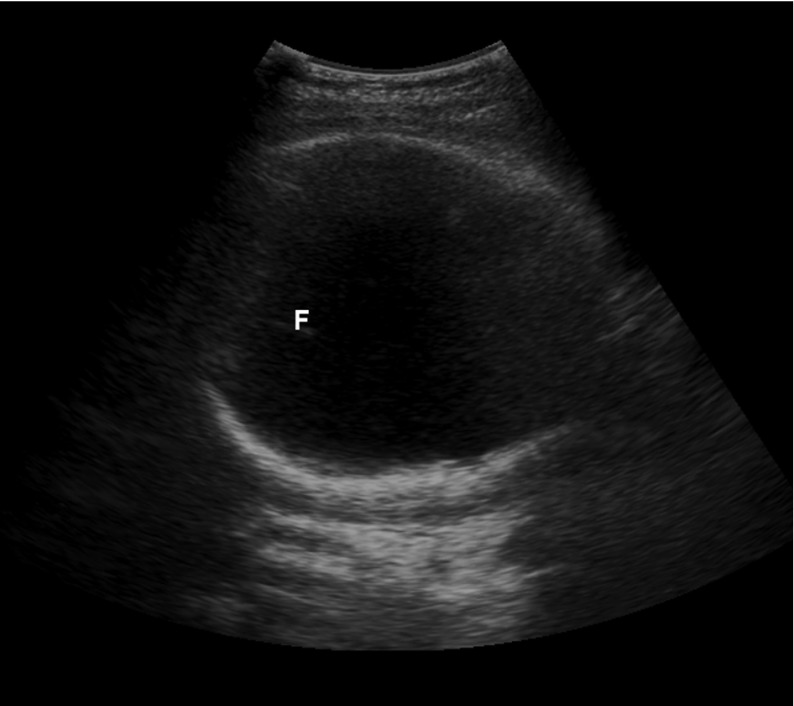
Prenatal ultrasound scans image of the fetus. Transabdominal transverse section of the fetal head shows disrupted falx (F).

Upon inspection, the obstetric ultrasound scan showed fluid filled cranial cavity with an absent cerebral cortex, thalami and basal ganglia. The third ventricle appeared to be dilated and the remnants of midbrain structures were present (see Figure 1). The cerebellum and other posterior fossa structures appeared to be normal. We noticed that the falx cerebri looked like it had been disrupted (see Figure 2). There was no polyhydramnios present and the umbilical artery doppler studies were normal. Sonographically, it was suggestive of hydranencephaly. The patient’s blood group was B positive and an infectious disease antibody test showed negative titers for rubella, HIV, Hepatitis B and toxoplasmosis. We chose to perform a caesarian section to avoid any obstetric complications that may arise from a possible cephalopelvic disproportion. The Pediatric team was informed about the situation and the parents were given advice and counseled with regards to the poor prognosis of the child’s abnormality. At week 39 of gestation, an emergency caesarian section was performed due to fetal distress. The newborn was a 2,980g female who had a normal physical appearance. The head of the newborn was of normal size (head circumference = 33.5 cm) but the head itself was particularly transilluminated.

**Figure 3.**
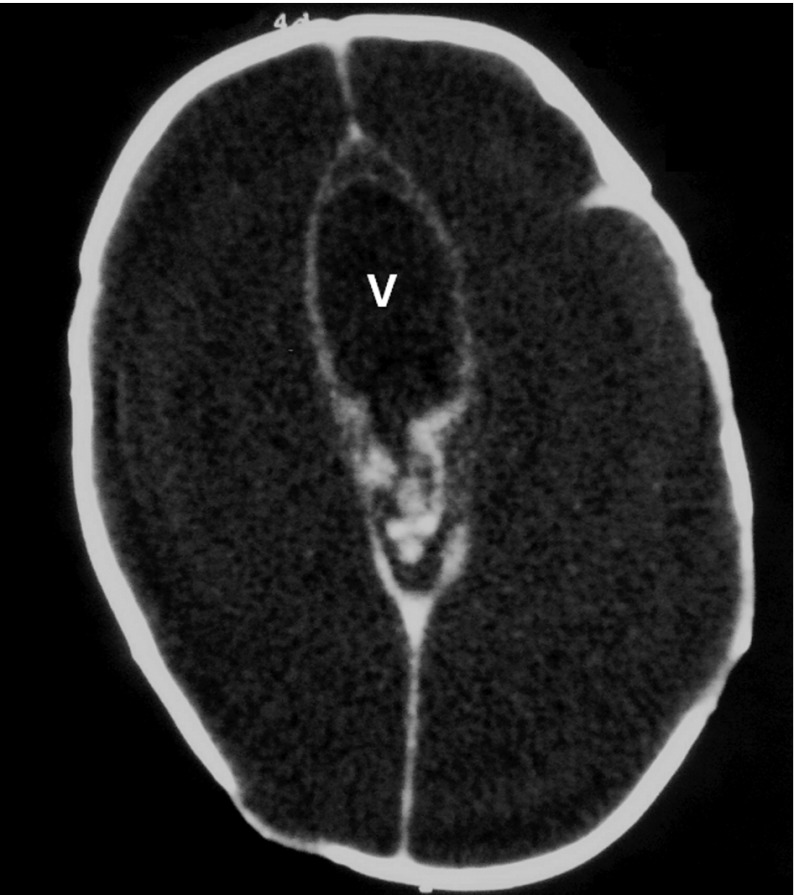
CT image of 2 days old newborns brain. Dilated third ventricle (V) with absent thalami and basal ganglia and cerebral cortex.

**Figure 4.**
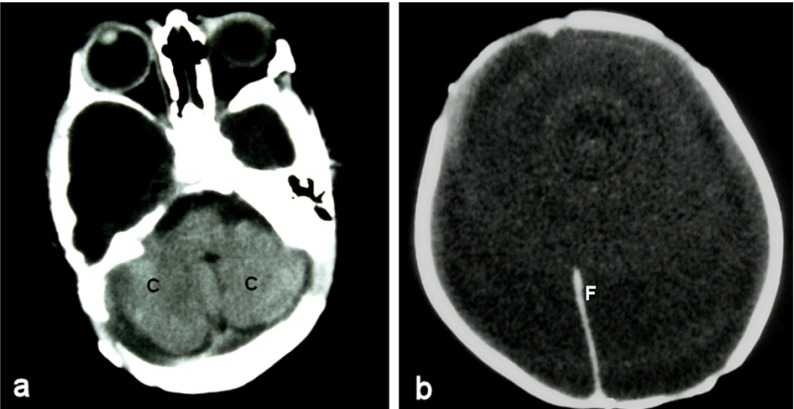
CT images of 2 days old newborns brain. ( **a**) Intact Cerebellum (C) and other posterior fossa structures. ( **b**) Shows disrupted falx cerebri (F).

The newborn was immediately transferred to the neonatal intensive care unit because of respiratory distress. A non-contrast computed tomography (CT) of the newborn’s head was performed two days after the delivery. The CT scan revealed that there was no cerebral cortex, thalami or basal ganglia. The third ventricle looked dilated and remnants of midbrain structures could be seen (see Figure 3). The cerebellum and other posterior fossa structures were preserved with disrupted falx cerebri (see Figure 4a, 4b). The CT scan therefore confirmed the prenatal diagnosis hydranencephaly. The baby died two weeks after birth due to cardiac arrest. The parents declined a postmortem examination.

## Discussion

Hydranencephaly is an encephaloclastic abnormality characterized by the absence and replacement of the cerebral hemispheres with cerebrospinal fluid and necrotic debris, covered by leptomeninges. Usually, there is no cerebral cortex but there may be partial preservation of a portion from the occipital lobe ^6^. The midbrain, thalamus, basal ganglia, choroids plexus, cerebellum and brain stem are usually preserved and contained within the skull. The falx cerebri is usually present but may be partially or entirely absent and the septum pellucidum may also be absent.

The etiopathogenesis of hydranencephaly is heterogeneous, and so several theories have been postulated for its occurrence. The most common etiology described is the occlusion of the supra-clinoid segment of the bilateral internal carotid arteries causing ischemic degeneration of structures supplied by them ^7^. Myers described the etiology of hydranencephaly by experimenting on laboratory monkeys. In his study, monkey fetuses were subjected to ligation of the bilateral carotid arteries and jugular veins in the neck at different gestational ages. These fetuses were then restored to the uterus, brought to term and subsequently delivered. Examination of the baby monkey brains revealed hydranencephaly, which resulted from a vascular shutdown predominantly when carried out during earlier gestational age ^8^.

Some case reports suggest the occlusion of the internal carotid arteries due to a temporary spasm rather that direct occlusion leading to ischemic destruction of certain brain structures ^9^. Other etiologies of hydranencephaly include intrauterine infections, which leads to the local destruction of brain tissue e.g. congenital toxoplasmosis ^10^ or other viral infections (adenovirus, cytomegalovirus, enterovirus, Epstein-Barr virus, herpes simplex virus, parvovirus, and respiratory syncytial viruses) ^3, 6, 11^. Another etiology that has arisen is the maternal exposure to carbon monoxide or butane gas, which can result in fetal hypoxia which in turn leads to massive tissue necrosis with cavitations, resorption of necrotized tissue and necrotizing vasculitis ^3, 12^.

Monozygotic twinning has also been associated with congenital hydranencephaly ^13^. It has been revealed that vascular interchanges between monozygotic twinning can result in the transfer of intravascular coagulation materials from the deceased co-twin to the surviving twin causing thromboembolism ^14, 15^. Hydranencephaly has also been associated with various congenital anomalies, including Fowler syndrome, arthrogryposis, renal aplastic dysplasia, poly-valvular heart defect, and trisomy ^17, 18^.

The cranial ultrasonographic imaging of hydranencephaly shows a large cystic mass filling the entire intracranial cavity with the absence or discontinuity of the cerebral cortex ^19^. The appearance of the thalami and brainstem protruding into the cystic cavity was characteristic, together with a midline echo from the remnants of the falx, the tentorium cerebelli and cerebellum ^19, 20^. The third ventricle and choroids plexus were often visible and the absence of the septum pellucidum may give rise to a single ventricle in the midline ^21^. There is a main difference in the diagnosis of extreme hydrocephaly, alobar holoprosencephaly and porencephaly ^22, 23^. In these conditions, the above-mentioned structures will still be surrounded by a rim of cortex; as they carry a better prognosis, it is essential to try and differentiate them in the ultrasonographic imaging of hydranencephaly. In extreme cases of hydrocephalus, the thin cortical layer may be difficult to recognize sonographically, and thus Magnetic resonance imaging (MRI) or intrauterine CT scans can be used to support the diagnosis ^24^.

The majority of cases of hydranencephaly are detected in the second half of a pregnancy ^18^; however there have been some cases of sonographic diagnosis of fetal hydranencephaly in the first trimester ^25^. Hydranencephaly has a poor prognosis, as the majority of brainstem functions are missing. Affected newborns can die at birth, but most infants die within the first year of their life ^26^. If a child does survive they will inevitably be severely handicapped.

The social and emotional problems that occur after a delivery of a child suffering from hydranencephaly can be terribly depressing for the family ^27^. Counseling the parents regarding the poor prognosis and the potential management options is advised to help them prepare for the potential outcome. Education of the prognosis is a necessary step that allows the family time to prepare and come to terms with the eventualities and can help provide immediate support when required ^27^. Because of the poor prognosis, termination of pregnancy is recommended once a definitive diagnosis has been established ^24^. If macrocrania is identified in late pregnancy then cephalocentesis may be suggested as an option to aid the delivery ^24^.

## Conclusion

The ultrasonographic findings that we described, led to the diagnosis of hydranencephaly being the most likely outcome and this was confirmed by postnatal CT of the fetal head. Remnant or preserved midbrain structures were observed in the most reported cases of hydranencephaly ^28, 29^ with the thalamus or basal ganglia (or both) structures preserved in the majority of cases. This is the first published case of prenatal diagnosed hydranencephaly with the absence of the thalami and basal ganglia along with ruminants of the midbrain in the same patient. Sonographic assessment is sufficient for the prenatal diagnosis of hydranencephaly in most cases, and MRI or an intrauterine CT should be used to support the sonographcic asssment and shouldn’t be considered as a first-line diagnostic tool.

Timely diagnosis is crucial in hydranencephaly cases as early treatment options can avoid obstetrics complications, and early diagnosis is particularly useful for anticipating the need to give appropriate counseling to the parents during the pregnancy. An early diagnosis is also fundamental for preparing the optimal conditions of delivery and allowing for a specialized pediatric delivery unit to be on hand for the delivery.

## Consent

Written informed consent obtained from the patient for publication of this case report and any accompanying images. A copy of the written consent is available for review by the Editors.

## References

[1] Raybaud C: Destructive lesion of the brain. *Neuroradiology.* 1983;25(4):265–291. 10.1007/BF00540238 6605492

[2] Crome L Sylvester PE: Hydranencephaly (hydrencephaly). *Arch Dis Child.* 1958;33(169):235–245. 10.1136/adc.33.169.235 13545875 PMC2012230

[3] Kurtz AB Johnson PT: Diagnosis please. Case 7: Hydranencephaly. *Radiology.* 1999;210(2):419–22. 10207424 10.1148/radiology.210.2.r99fe50419

[4] Halsey JH: Hydranencephaly. In *Handbook of Clinical Neurology* *Malformations* Edited by PJ Vinken, GW Bruyn and JL Klawans. Amsterdam: Elsevier;1987;50:337–353.

[5] Malheiros JA Trivelato FP Oliveira MM: Endoscopic choroid plexus cauterization versus ventriculoperitoneal shunt for hydranencephaly and near hydranencephaly: a prospective study. *Neurosurgery.* 2010;66(3):459–64. 10.1227/01.NEU.0000365264.99133.CA 20173541

[6] Chinsky JM: Hydranencephaly: Transillumination May Not Illuminate Diagnosis. *NeoReviews.* 2012;13:e233–e240 10.1542/neo.13-4-e233

[7] Kelly TG Sharif UM Southern JF: An unusual case of hydranencephaly presenting with an anterior midline cyst, a posterior calcified mass, cerebellar hypoplasia and occlusion of the posterior cerebral arteries. *Pediatr Radiol.* 2011;41(2):274–7. 10.1007/s00247-010-1894-1 21104240

[8] Myers RE: Brain pathology following fetal vascular occlusion: an experimental study. *Invest Ophthalmol.* 1969;8(1):41–50. 4974267

[9] Lindenberg R Swanson PD: “Infantile Hydranencephaly”, a report of five cases of infarction of both cerebral hemispheres in infancy. *Brain.* 1967;90(4):839–850. 10.1093/brain/90.4.839 6075814

[10] Altshuler G: Toxoplasmosis as a cause of hydranencephaly. *Am J Dis Child.* 1973;125(2):251–252. 10.1001/archpedi.1973.04160020073014 4568341

[11] Christie JD Rakusan TA Martinez MA: Hydranencephaly caused by congenital infection with herpes simplex virus. *Pediatr Infect Dis.* 1986;5(4):473–8. 10.1097/00006454-198607000-00020 3725658

[12] Fernàndez F Pèrez-Higueras A Hernàndez R: Hydranencephaly after maternal butane-gas intoxication during pregnancy. *Dev Med Child Neurol.* 1986;28(3):361–363. 3721079 10.1111/j.1469-8749.1986.tb03885.x

[13] Jung Jack H Graham JM Jr Schultz N: Congenital hydranencephaly/porencephaly due to vascular disruption in monozygotic twins. *Pediatrics.* 1984;73(4):467–469. 6709425

[14] Szymonowicz W Preston H Yu VY: The surviving monozygotic twin. *Arch Dis Child.* 1986;61(5):454–458. 10.1136/adc.61.5.454 3717990 PMC1777779

[15] Larroche JC Droullé P Delezoide AL: Brain damage in monozygous twins. *Biol Neonate.* 1990;57(5):261–278. 10.1159/000243201 2182133

[16] Usta IM AbuMusa AA Khoury NG: Early ultrasonographic changes in Fowler syndrome features and review of the literature. *Prenat Diagn.* 2005;25(11):1019–23. 10.1002/pd.1240 16231307

[17] Bendon RW Siddigi T de Courten-Myers G: Recurrent developmental anomalies: 1. Syndrome with hidranencephaly with renal aplastic dysplasia; 2. Polyvalvular developmental heart defect. *Am J Med Genet Suppl.* 1987;3:357–365. 10.1002/ajmg.1320280541 3130870

[18] Alasdair GW: Hunter: Brain. In *Human malformations and related anomalies* 2 ^nd^Edition. Edited by Roger E Stevenson, Judith G Hall. New York: Oxford University Press;2006;639–645.

[19] Eisenberg RL: Clinical imaging: an atlas of differential diagnosis 5 ^th^ ed.Philadelphia, Lippincott Williams & Wilkins,1996;1419 Reference Source

[20] David KJ Philip JS Carl PW: High Risk Pregnancy: Management Options. 3rd Edition.Pennsylvania: Elsevier;2006;386–387 10.1576/toag.9.3.211.27345

[21] Gordon IRS Ross FGM: Diagnostic radiology in pediatrics. Illustrated edition.Michigan: Butterworths;1977;305–306.

[22] Nyberg DA McGahan JP Pretorius DH: Diagnostic imaging of fetal anomalies.Philadelphia: Lippincott Williams & Wilkins;2003;269 Reference Source

[23] Joshua Copel: Obstetric Imaging: Expert Radiology series.Elsevier;2012;238.

[24] Keith CD Hylton BM David OC: Ultrasound in obstetrics and gynecology. 2 ^nd^ Edition.Michigan: Churchill Livingstone;2001;326–327.

[25] Lin YS Chang FM Liu CH: Antenatal detection of hydranencephaly at 12 weeks, menstrual age. *J Clin Ultrasound.* 1992;20(1):62–4. 10.1002/jcu.1870200112 1309546

[26] Hadi HA Mashini IS Devoe LD: Ultrasonographic prenatal diagnosis of hydranencephaly. A case report. *J Reprod Med.* 1986;31(4):254–6. 3519967

[27] Petridis AK Ulf RK Alexandros D: Delayed diagnosis of hydranencephaly in a nine-month-old child. *Clinics and Practice.* 2011;1(3):e65 Reference Source 24765326 10.4081/cp.2011.e65 PMC3981365

[28] Aylward GP Anthony L John M: Behavioral and neurological characteristics of a hydranencephalic infant. *Dev Med Child Neurol.* 1978;20(2):211–217. 10.1111/j.1469-8749.1978.tb15206.x 640266

[29] Hamby WB Krauss RF Beswick WF: Hydranencephaly: Clinical Diagnosis Presentation of Seven Cases. *Pediatrics.* 1950;6(3):371–383. 14780790

