# Supplementary material for: A rare variation of hydranencephaly: case report
Source: F1000Res. 2014 Jan 30;1:22. Originally published 2012 Oct 3. [Version 3] doi: 10.12688/f1000research.1-22.v3 (PMC3945765; doi:10.12688/f1000research.1-22.v3)
Supplement: Peer review history

# Peer review: Referee response for version 3

**DOI:** 10.5256/f1000research.3782.r4020

**Published:** 2014-03-10

**Authors:** Jeffrey Chinsky

**Affiliations:** 1 Department of Pediatrics, Johns Hopkins University School of Medicine, Baltimore, MD, USA

There are three typographical errors in the Conclusion -

- Line 1, remove the comma after "....described, ..."
- Line 3, remove "the" in "observed in the most reported...."
- Line 7, the correct spelling is "sonographic", not "sonogrpahcic"

Otherwise, I approve of the latest version.

I have read this submission. I believe that I have an appropriate level of expertise to confirm that it is of an acceptable scientific standard.

# Peer review: Referee response for version 3

**DOI:** 10.5256/f1000research.3782.r3454

**Published:** 2014-01-31

**Authors:** Athanasios Petridis

**Affiliations:** 1 Department of Neurosurgery, University of Schleswig-Holstein, Kiel, Germany

I have read this submission. I believe that I have an appropriate level of expertise to confirm that it is of an acceptable scientific standard.

# Peer review: Referee response for version 2

**DOI:** 10.5256/f1000research.1228.r3215

**Published:** 2014-01-21

**Authors:** Jeffrey Chinsky

**Affiliations:** 1 Department of Pediatrics, Johns Hopkins University School of Medicine, Baltimore, MD, USA

This second revision is fine except for the following suggestions/corrections:

1. Reference 6 should read Chinsky JM, not Jeffrey MC.
2. In the introduction I would suggest changing “ *significantly higher prognosis*” (line 6) to “potentially better prognosis”.
3. In the Discussion, paragraph 6; remove "really" from " *Hydranencephaly has a really poor prognosis*" so that it simply reads "poor prognosis".

I have read this submission. I believe that I have an appropriate level of expertise to confirm that it is of an acceptable scientific standard.

# Peer review: Referee response for version 2

**DOI:** 10.5256/f1000research.1228.r750

**Published:** 2013-02-04

**Authors:** Athanasios Petridis

**Affiliations:** 1 Department of Neurosurgery, University of Schleswig-Holstein, Kiel, Germany

The corrections made by the author are very satisfying. I now approve this article.

I have read this submission. I believe that I have an appropriate level of expertise to confirm that it is of an acceptable scientific standard.

# Peer review: Referee response for version 1

**DOI:** 10.5256/f1000research.118.r305

**Published:** 2012-11-14

**Authors:** Athanasios Petridis

**Affiliations:** 1 Department of Neurosurgery, University of Schleswig-Holstein, Kiel, Germany

This case report is interesting. Except of some grammatical mistakes, I do have one suggestion.

The authors should also discuss the social aspects and the social stress which accompanies families with hydranencephalic children. It is important to induce abortion when possible in such cases since the prognosis is very poor. Early diagnosis is the most important aspect in such cases. The paper of mine, Petridis et al. (2011) deals with the social aspects of hydranencephaly and should be cited by the author.

I have read this submission. I believe that I have an appropriate level of expertise to confirm that it is of an acceptable scientific standard, however I have significant reservations, as outlined above.

# Response

**Published:** 2013-01-31

**Authors:** Buddhika Wijerathne

**Affiliations:** Faculty of Medicine and Allied Scinces,Rajarata University of Sri Lanka,Sri Lanka, Sri Lanka

We would like to thank Dr Petridis for the valuable time spent reviewing our manuscript and the important comments he has made. We have attempted to address and incorporate most of the concerns that were raised in version 2 of our article.

# Peer review: Referee response for version 1

**DOI:** 10.5256/f1000research.118.r304

**Published:** 2012-11-12

**Authors:** Jeffrey Chinsky

**Affiliations:** 1 Department of Pediatrics, Johns Hopkins University School of Medicine, Baltimore, MD, USA

This case report presents an interesting case of apparent hydranencephalic changes, in addition to more extensive developmental brain anomalies (lack of thalamus and basal ganglia). However, including the points made below would make the manuscript much more useful as an educational resource:

The manuscript would be enhanced if the author’s did more than just itemize a list of associated etiologies for hydranancephaly. The authors should provide a paragraph explaining how any of these etiologies could specifically produce the anomalies observed (cerebral arterial stroke or spasm of which specific vessels) and perhaps contrast it with any syndromic or associated genomic alterations listings of similar brain findings (or at least provide evidence that they researched this aspect).

I’m not sure all would accept this as a variant of the same processes that cause classic hydranencephaly since it appears to be so much more extensive anomalous formation of the final fetal brain. The authors may have to do a little more research and discussion to support their contention based on how others who have written about hydranencphaly and define the condition.

There are several grammatical errors (e.g. in the Abstract “such that absent cortex” is an incomplete phrase to end the sentence) which should be addressed, and I suggest the article is proof read again to ensure it reads how a published manuscript should do.

I have read this submission. I believe that I have an appropriate level of expertise to confirm that it is of an acceptable scientific standard.

# Response

**Published:** 2013-01-31

**Authors:** Buddhika Wijerathne

**Affiliations:** Faculty of Medicine and Allied Scinces,Rajarata University of Sri Lanka,Sri Lanka, Sri Lanka

We would like to thank the reviewer, Dr. Chinsky for the valuable time spent reviewing our manuscript and the important comments he has made. We have attempted to address and incorporate most of the concerns that were raised in version 2 of our article.
